# Author Correction: Unwinding of a eukaryotic origin of replication visualized by cryo-EM

**DOI:** 10.1038/s41594-024-01398-0

**Published:** 2024-09-09

**Authors:** Sarah S. Henrikus, Marta H. Gross, Oliver Willhoft, Thomas Pühringer, Jacob S. Lewis, Allison W. McClure, Julia F. Greiwe, Giacomo Palm, Andrea Nans, John F. X. Diffley, Alessandro Costa

**Affiliations:** 1 Macromolecular Machines Laboratory, Francis Crick Institute, London, UK; 2 Chromosome Replication Laboratory, Francis Crick Institute, London, UK; 3 Structural Biology Science Technology Platform, Francis Crick Institute, London, UK; 4 Present Address: Genome Stability Unit, St. Vincent’s Institute of Medical Research, Fitzroy, Victoria Australia; 5 Present Address: Department of Biochemistry and Molecular Genetics, University of Colorado Anschutz Medical Campus, Aurora, CO USA

**Keywords:** Chromosomes, Cryoelectron microscopy, DNA replication, Replisome

Correction to: *Nature Structural & Molecular Biology* 10.1038/s41594-024-01280-z, published online 17 May 2024.

In the version of the article initially published, the text “Composite map showing sCMGE (apix = 1.08 sigma = 0.00828) and additional density observed after particle binning and Pol epsilon signal subtraction (apix = 2.16, sigma = 0.0157)” was missing from the legend to Fig. 2d and has now been added to the HTML and PDF versions of the article.

