# Supplementary material for: Unwinding of a eukaryotic origin of replication visualized by cryo-EM
Source: Nat Struct Mol Biol. 2024 May 17;31(8):1265–76. doi: 10.1038/s41594-024-01280-z (PMC11327109; doi:10.1038/s41594-024-01280-z)
Supplement: Supplementary file 1 — Supplementary Tables 1–3 and Supplementary Information (DNA sequence of pSSH006) [file 41594_2024_1280_MOESM1_ESM.pdf]

nature structural & molecular biology

Article https://doi.org/10.1038/s41594-024-01280-z

Unwinding of a eukaryotic origin of replication visualized by cryo-EM

In the format provided by the authors and unedited

Supplementary Information

Supplementary Table 1: Oligonucleotides and gene blocks used in this study.

Name	Usage	Sequence	Reference
oMG40	Forward primer to generate pMG73 plasmid	GCTTCAATTGTATAATCACCTCCTG CCGCAGCTGCCACAACCGCAGCTG TTAAACCAGC	This study.
oMG41	Reverse primer to generate pMG73 plasmid	GCTGGTTTAACAGCTGCGGTTGTG GCAGCTGCGGCAGGAGGTGATTAT ACAATTGAAGC	This study.
oJL019	Forward primer for generation of 168bp M.HpaII ARS1	ATATATCC*GGCCTGTATTTTACAG ATTTTATGT TTAGATCTTTTATGC	(Miller, TM *et al.* Nature, 2019; Lewis, JS *et al.* Nature 2021)
oJL040	Reverse primer for generation of 168bp M.HpaII ARS1	AGGGCGCC*GGAACTGGGAAAATA GCAAATTTCG TCAAAAATGC	(Miller, TM *et al.* Nature, 2019; Lewis, JS *et al.* Nature 2021)

Supplementary Table 2: DNA plasmids used in this study.

Plasmid	Characteristics	Usage	Selection	Reference
pAM3	GST-Cdc6 in pGEX-6p-1	Cdc6 purification	Amp	(Frigola, J *et al.* Nature 2013)
pJL003	pTwinStrepII-GINS with TwinStrepII tag on N-terminus of Psf3	GINS purification (used in EM assays)	Kan	(Lewis, JS *et al.* Nature 2021)
pFJD5	pHis-GINS with His tag on N-terminus of Psf3	GINS purification (used for DNA replication assay and EM assays involving Rad53)	Kan	(Gambus, *et al.* EMBO J. 2009, Yeeles, JTP *et al.* Nature 2015)
pGC441	pGST-Sdl2	Sld2 purification	AMP	(Posse, V *et al.* Methods Enzymol. 2021)
pMD132	pHis-Mcm10-Flag	Mcm10 purification	Kan	(Douglas, ME *et al.* Nature 2018)
pSSH006	pHis-Mcm10$^{D348-571}$-Flag	Mcm10-CTD purification	Amp	This study.
pJL004	pTwinStrepII-M.HpaII	M.HpaII purfication	Amp	(Lewis, JS *et al.* Nature 2021)

pET-RAD53	pET-Rad53-His	Rad53 purification		(Gilbert, CS *et al.* Mol. Cell, 2001)
pAWM7	pET21b-Rad53 ^{K227A,D339A} -His	Rad53^{K227A,D339A} (KD) purification	Amp	(McClure, AW, *et al.* eLife 2021)
pJF4	pRS305/Mcm6/Mcm7	To obtain pMG73 plasmid	Amp Leu2	(Frigola, J *et al.* Nature 2013)
pMG73	pRS305/Mcm6 4A, Mcm7	To obtain yMG44 strain for Mcm6 4A expression)	Amp Leu2	This study.
pJY22	10.6 kb plasmid DNA with ARS1	DNA replication assay	Amp	(Yeeles, JTP *et al.* Mol. Cell 2017)

Supplementary Table 3: Yeast constructs

Strain	Genotype	Usage	Reference
ySD-ORC	*MAT**a** ade2-1 ura3-1 his3-11,15 trp1-1 leu2-3,112 can1-100 bar1::Hyg pep4::KanMX his3::HIS3pRS303/ORC3, ORC4 trp1::TRP1pRS304/ORC5, ORC6 ura3::URA3pRS306/CBP-ORC1, ORC2*	ORC purification	(Frigola, J *et al.* Nature 2013)
yAM33	*MAT**a** ade2-1 ura3-1 his3-11,15 trp1-1 leu2-3,112 can1-100 bar1::Hyg pep4::KanMX his3::HIS3pRS303/CDT1,GAL4 trp1::TRP1pRS304/MCM4,MCM5 leu2::LEU2pRS305/MCM6 MCM7 ura3::URA3pRS306/MCM2,CBP-MCM3*	Mcm2–7/Cdt1 purification	(Coster, G *et al.* Mol. Cell 2014)
ySDK8	*MAT**a** ade2-1 ura3-1 his3-11,15 trp1-1 leu23,112 can1-100 bar1::Hyg pep4::KanMX trp1::TRP1pRS304/CDC7, CBP-DBF4*	DDK purification	(On, KF *et al.* EMBO J. 2014)
yAE88	*MAT**a** ade2-1 ura3-1 his3-11,15 trp1-1 leu2-3,112 can1-100 bar1::Hyg pep4::KanMX his3::HIS3pRS303/CBP-TEV-CLB5(D1-100), GAL4 ura3::URA3pRS306/CKS1, CDC28*	CDK purification	(Hill, J *et al.* bioRxiv 2020)
yJY13	*MAT**a** ade2-1 ura3-1 his3-11,15 trp1-1 leu2-3,112 can1-100 bar1::Hyg*	Cdc45 purification	(Yeeles, JTP *et al.* Nature 2015)

	pep4::KanMX<br>*his3::HIS3pRS303/Cdc45iflag2, GAL4*		
yJY26	*MAT**a** ade2-1 ura3-1 his3-11,15 trp1-1 leu2-3,112 can1-100*<br>*bar1::Hyg*<br>*pep4::KanMX*<br>*his3::HIS3pRS303/Dpb11-3xflag (Nat-NT2), GAL4*	Dpb11 purification	(Yeeles, JTP *et al*. Nature 2015)
yAJ2	*MAT**a** ade2-1 ura3-1 his3-11,15 trp1-1 leu2-3,112 can1-100*<br>*bar1::Hyg*<br>*pep4::KanMX*<br>*ura3::URA3pRS306/Dpb2, Dpb3*<br>*trp1::TRP1pRS304/Pol2, Dpb4-Tev-CBP*	Pol epsilon purification	(Yeeles, JTP *et al*. Nature 2015)
yTD6	*MAT**a** ade2-1 ura3-1 his3-11,15 trp1-1 leu2-3,112 can1-100*<br>*bar1::Hyg*<br>*pep4::KanMZ*<br>*his3::HIS3pRS303/SLD3-TCP, GAL4,*<br>*leu2::LEU2pRS305/SLD7*	Sld3 –7 purification	(Yeeles, JTP *et al*. Nature 2015)
yAE95	*MAT**a** ade2-1 ura3-1 his3-11,15 trp1-1 leu2-3,112 can1-100,*<br>*bar1::Hyg*<br>*pep4::KanMX*<br>*trp1::TRP1pRS304/POL1, POL12,*<br>*ura3::URA3pRS306/CBP-TEV-PRI1, PRI2*	Pol alpha purification	(Hill, J *et al*. bioRxiv 2020)
yAE42	*MAT**a** ade2-1 ura3-1 his3-11,15 trp1-1 leu2-3,112 can1-100*<br>*bar1::Hyg*<br>*pep4::KanMX*<br>*his3::HIS3pRS303/CBP-TOP1+Gal4*	TopoI purification	(Yeeles, JTP *et al*. Mol. Cell 2017)
yAE31	*MAT**a** ade2-1 ura3-1 his3-11,15 trp1-1 leu2-3,112 can1-100*<br>*bar1::Hyg*<br>*pep4::KanMX*<br>*his3::HIS3pRS303/CBP-TEV-RFA1, GAL4,*<br>*ura3::URA3pRS306/RFA2, RFA3*	RPA purification	(Yeeles, JTP *et al*. Nature 2015)
yAE160	*MAT**a** ade2-1 ura3-1 his3-11,15 trp1-1 leu2-3,112 can1-100*<br>*bar1::Hyg*<br>*pep4::KanMX*<br>*trp1::TRP1pRS304Mcm4, Mcm5*<br>*ura3::URA3pRS306/Mcm2, CBP-TEV Mcm3 his3::HIS3pRS303/Cdt1,Gal4*<br>*leu2::LEU2pRS305/Mcm6 2E, Mcm7*	Mcm6 2E mutant purification	(Lewis, JS *et al*. Nature 2021)
yJY32	*MAT**a** ade2-1 ura3-1 his3-11,15 trp1-1 leu2-3,112 can1-100*<br>*bar1::Hyg*<br>*pep4::KanMX*<br>*his3::HIS3pRS303-Mrc1 C-term 2x Flag*	Mrc1 purification	(Yeeles, JTP *et al*. Mol. Cell 2017)

yAM20	*MAT***a** *ade2-1 ura3-1 his3-11,15 trp1-1 leu2-3,112 can1-100 bar1::Hyg pep4::KanMX trp1::TRP1pRS304Mcm4, Mcm5 ura3::URA3pRS306/Mcm2, CBP-TEV Mcm3 his3::HIS3pRS303/Cdt1,Gal4*	Background strain	(Coster, G *et al*. Mol. Cell 2014)
yMG44	*MAT***a** *ade2-1 ura3-1 his3-11,15 trp1-1 leu2-3,112 can1-100 bar1::Hyg pep4::KanMX trp1::TRP1pRS304Mcm4, Mcm5 ura3::URA3pRS306/Mcm2, CBP-TEV Mcm3 his3::HIS3pRS303/Cdt1,Gal4 leu2::LEU2pRS305/Mcm6 4A, Mcm7*	To overexpress Mcm6 4A (Mcm6 R614A D615A E616A E617A)	This study.

Supplementary information

DNA Sequence of pSSH006:

GATCTCGATCCCGCGAAATTAATACGACTCACTATAGGGGAATTGTGAGCGGAT
AACAATTCCCCTCTAGAAATAATTTTGTTTAACTTTAAGAAGGAGATATACATA
TGCATCATCATCATCATCACGTGAATTCGATGAATGATCCTCGTGAAATTTTAGC
GGTTGATCCGTACAATAATATTACTTCTGATGAAGAGGATGAGCAAGCCATCGC
GAGAGAACTTGAATTTATGGAACGAAAGAGGCAGGCCTTAGTGGAACGATTAAA
AAGAAAGCAAGAATTTAAGAAACCCCAGGATCCTAATTTTGAAGCCATCGAGGT
ACCTCAATCTCCTACCAAAAACCGTGTGAAAGTGGGGTCTCATAATGCTACACAA
CAAGGCACAAAATTCGAAGGTTCGAATATTAATGAAGTAAGGTTATCTCAATTAC
AGCAGCAACCAAAACCACCAGCTAGTACAACCACATACTTTATGGAGAAATTTC
AAAACGCAAAGAAGAACGAAGATAAACAAATTGCCAAGTTTGAAAGCATGATG
AATGCAAGAGTACATACGTTCAGTACCGATGAGAAGAAATATGTGCCGATAATC
ACAAACGAATTAGAAAGCTTTTCAAATCTTTGGGTTAAAAAGAGGTACATACCTG
AAGATGACTTAAAACGGGCTTTGCATGAGATCAAAATCCTTCGGTTGGGCAAAC
TTTTTGCTAAAATTCGCCCACCTAAATTTCAAGAGCCTGAATACGCCAACTGGGC
CACCGTAGGCCTCATTAGCCACAAATCGGACATCAAATTTACATCATCTGAAAAG
CCAGTCAAATTCTTCATGTTCACCATAACGGACTTTCAGCATACACTAGATGTTT
ATATCTTCGGGAAAAAGGGTGTAGAAAGATATTATAATCTTCGCCTGGGTGATGT

GATAGCAATATTAAACCCAGAAGTACTACCATGGAGACCCTCAGGGCGAGGAAA
TTTTATCAAATCCTTCAACCTTCGAATTAGTCATGACTTCAAATGTATCCTGGAGA
TAGGTTCAAGTAGAGATTTAGGTTGGTGTCCCATAGTGAATAAAAAGACTCACA
AAAAATGTGGCTCTCCCATTAACATATCTCTTCATAAGTGTTGCGATTACCATAG
AGAAGTGCAATTTCGTGGAACAAGTGCTAAAAGAGGTGGTTCTGGTGACTACAA
AGACCATGACGGTGATTATAAAGATCATGACATCGATTACAAGGATGACGATGA
CAAGTAAGAATTCGCTCGAGATCGATGATATTCGAGCCTAGGTATAATCGGATCC
GGCTGCTAACAAAGCCCGAAAGGAAGCTGAGTTGGCTGCTGCCACCGCTGAGCA
ATAACTAGCATAACCCCTTGGGGCCTCTAAACGGGTCTTGAGGGGTTTTTTGCTG
AAAGGAGGAACTATATCCGGATATCCCGCAAGAGGCCCGGCAGTACCGGCATAA
CCAAGCCTATGCCTACAGCATCCAGGGTGACGGTGCCGAGGATGACGATGAGCG
CATTGTTAGATTTCATACACGGTGCCTGACTGCGTTAGCAATTTAACTGTGATAA
ACTACCGCATTAAAGCTAGCTTATCGATGATAAGCTGTCAAACATGAGAATTAAT
TCTTGAAGACGAAAGGGCCTCGTGATACGCCTATTTTTATAGGTTAATGTCATGA
TAATAATGGTTTCTTAGACGTCAGGTGGCACTTTTCGGGGAAATGTGCGCGGAAC
CCCTATTTGTTTATTTTTCTAAATACATTCAAATATGTATCCGCTCATGAGACAAT
AACCCTGATAAATGCTTCAATAATATTGAAAAAGGAAGAGTATGAGTATTCAAC
ATTTCCGTGTCGCCCTTATTCCCTTTTTTGCGGCATTTTGCCTTCCTGTTTTTGCTC
ACCCAGAAACGCTGGTGAAAGTAAAAGATGCTGAAGATCAGTTGGGTGCACGAG
TGGGTTACATCGAACTGGATCTCAACAGCGGTAAGATCCTTGAGAGTTTTCGCCC
CGAAGAACGTTTTCCAATGATGAGCACTTTTAAAGTTCTGCTATGTGGCGCGGTA
TTATCCCGTGTTGACGCCGGGCAAGAGCAACTCGGTCGCCGCATACACTATTCTC
AGAATGACTTGGTTGAGTACTCACCAGTCACAGAAAAGCATCTTACGGATGGCA
TGACAGTAAGAGAATTATGCAGTGCTGCCATAACCATGAGTGATAACACTGCGG
CCAACTTACTTCTGACAACGATCGGAGGACCGAAGGAGCTAACCGCTTTTTTGCA
CAACATGGGGGATCATGTAACTCGCCTTGATCGTTGGGAACCGGAGCTGAATGA
AGCCATACCAAACGACGAGCGTGACACCACGATGCCTGCAGCAATGGCAACAAC
GTTGCGCAAACTATTAACTGGCGAACTACTTACTCTAGCTTCCCGGCAACAATTA
ATAGACTGGATGGAGGCGGATAAAGTTGCAGGACCACTTCTGCGCTCGGCCCTT
CCGGCTGGCTGGTTTATTGCTGATAAATCTGGAGCCGGTGAGCGTGGGTCTCGCG
GTATCATTGCAGCACTGGGGCCAGATGGTAAGCCCTCCCGTATCGTAGTTATCTA
CACGACGGGGAGTCAGGCAACTATGGATGAACGAAATAGACAGATCGCTGAGAT
AGGTGCCTCACTGATTAAGCATTGGTAACTGTCAGACCAAGTTTACTCATATATA
CTTTAGATTGATTTAAAACTTCATTTTTAATTTAAAAGGATCTAGGTGAAGATCCT

TTTTGATAATCTCATGACCAAAATCCCTTAACGTGAGTTTTCGTTCCACTGAGCGT
CAGACCCCGTAGAAAAGATCAAAGGATCTTCTTGAGATCCTTTTTTTCTGCGCGT
AATCTGCTGCTTGCAAACAAAAAAACCACCGCTACCAGCGGTGGTTTGTTTGCCG
GATCAAGAGCTACCAACTCTTTTTCCGAAGGTAACTGGCTTCAGCAGAGCGCAG
ATACCAAATACTGTCCTTCTAGTGTAGCCGTAGTTAGGCCACCACTTCAAGAACT
CTGTAGCACCGCCTACATACCTCGCTCTGCTAATCCTGTTACCAGTGGCTGCTGC
CAGTGGCGATAAGTCGTGTCTTACCGGGTTGGACTCAAGACGATAGTTACCGGAT
AAGGCGCAGCGGTCGGGCTGAACGGGGGGTTCGTGCACACAGCCCAGCTTGGAG
CGAACGACCTACACCGAACTGAGATACCTACAGCGTGAGCTATGAGAAAGCGCC
ACGCTTCCCGAAGGGAGAAAGGCGGACAGGTATCCGGTAAGCGGCAGGGTCGG
AACAGGAGAGCGCACGAGGGAGCTTCCAGGGGGAAACGCCTGGTATCTTTATAG
TCCTGTCGGGTTTCGCCACCTCTGACTTGAGCGTCGATTTTTGTGATGCTCGTCAG
GGGGGCGGAGCCTATGGAAAAACGCCAGCAACGCGGCCTTTTTACGGTTCCTGG
CCTTTTGCTGGCCTTTTGCTCACATGTTCTTTCCTGCGTTATCCCCTGATTCTGTGG
ATAACCGTATTACCGCCTTTGAGTGAGCTGATACCGCTCGCCGCAGCCGAACGAC
CGAGCGCAGCGAGTCAGTGAGCGAGGAAGCGGAAGAGCGCCTGATGCGGTATTT
TCTCCTTACGCATCTGTGCGGTATTTCACACCGCAATGGTGCACTCTCAGTACAA
TCTGCTCTGATGCCGCATAGTTAAGCCAGTATACACTCCGCTATCGCTACGTGAC
TGGGTCATGGCTGCGCCCCGACACCCGCCAACACCCGCTGACGCGCCCTGACGG
GCTTGTCTGCTCCCGGCATCCGCTTACAGACAAGCTGTGACCGTCTCCGGGAGCT
GCATGTGTCAGAGGTTTTCACCGTCATCACCGAAACGCGCGAGGCAGCTGCGGT
AAAGCTCATCAGCGTGGTCGTGAAGCGATTCACAGATGTCTGCCTGTTCATCCGC
GTCCAGCTCGTTGAGTTTCTCCAGAAGCGTTAATGTCTGGCTTCTGATAAAGCGG
GCCATGTTAAGGGCGGTTTTTTCCTGTTTGGTCACTGATGCCTCCGTGTAAGGGG
GATTTCTGTTCATGGGGGTAATGATACCGATGAAACGAGAGAGGATGCTCACGA
TACGGGTTACTGATGATGAACATGCCCGGTTACTGGAACGTTGTGAGGGTAAAC
AACTGGCGGTATGGATGCGGCGGGACCAGAGAAAAATCACTCAGGGTCAATGCC
AGCGCTTCGTTAATACAGATGTAGGTGTTCCACAGGGTAGCCAGCAGCATCCTGC
GATGCAGATCCGGAACATAATGGTGCAGGGCGCTGACTTCCGCGTTTCCAGACTT
TACGAAACACGGAAACCGAAGACCATTCATGTTGTTGCTCAGGTCGCAGACGTTT
TGCAGCAGCAGTCGCTTCACGTTCGCTCGCGTATCGGTGATTCATTCTGCTAACC
AGTAAGGCAACCCCGCCAGCCTAGCCGGGTCCTCAACGACAGGAGCACGATCAT
GCGCACCCGTGGCCAGGACCCAACGCTGCCCGAGATGCGCCGCGTGCGGCTGCT
GGAGATGGCGGACGCGATGGATATGTTCTGCCAAGGGTTGGTTTGCGCATTCAC

AGTTCTCCGCAAGAATTGATTGGCTCCAATTCTTGGAGTGGTGAATCCGTTAGCG
AGGTGCCGCCGGCTTCCATTCAGGTCGAGGTGGCCCGGCTCCATGCACCGCGAC
GCAACGCGGGGAGGCAGACAAGGTATAGGGCGGCGCCTACAATCCATGCCAACC
CGTTCCATGTGCTCGCCGAGGCGGCATAAATCGCCGTGACGATCAGCGGTCCAAT
GATCGAAGTTAGGCTGGTAAGAGCCGCGAGCGATCCTTGAAGCTGTCCCTGATG
GTCGTCATCTACCTGCCTGGACAGCATGGCCTGCAACGCGGGCATCCCGATGCCG
CCGGAAGCGAGAAGAATCATAATGGGGAAGGCCATCCAGCCTCGCGTCGCGAAC
GCCAGCAAGACGTAGCCCAGCGCGTCGGCCGCCATGCCGGCGATAATGGCCTGC
TTCTCGCCGAAACGTTTGGTGGCGGGACCAGTGACGAAGGCTTGAGCGAGGGCG
TGCAAGATTCCGAATACCGCAAGCGACAGGCCGATCATCGTCGCGCTCCAGCGA
AAGCGGTCCTCGCCGAAAATGACCCAGAGCGCTGCCGGCACCTGTCCTACGAGT
TGCATGATAAAGAAGACAGTCATAAGTGCGGCGACGATAGTCATGCCCCGCGCC
CACCGGAAGGAGCTGACTGGGTTGAAGGCTCTCAAGGGCATCGGTCGAGATCCC
GGTGCCTAATGAGTGAGCTAACTTACATTAATTGCGTTGCGCTCACTGCCCGCTT
TCCAGTCGGGAAACCTGTCGTGCCAGCTGCATTAATGAATCGGCCAACGCGCGG
GGAGAGGCGGTTTGCGTATTGGGCGCCAGGGTGGTTTTTCTTTTCACCAGTGAGA
CGGGCAACAGCTGATTGCCCTTCACCGCCTGGCCCTGAGAGAGTTGCAGCAAGC
GGTCCACGCTGGTTTGCCCCAGCAGGCGAAAATCCTGTTTGATGGTGGTTAACGG
CGGGATATAACATGAGCTGTCTTCGGTATCGTCGTATCCCACTACCGAGATATCC
GCACCAACGCGCAGCCCGGACTCGGTAATGGCGCGCATTGCGCCCAGCGCCATC
TGATCGTTGGCAACCAGCATCGCAGTGGGAACGATGCCCTCATTCAGCATTTGCA
TGGTTTGTTGAAAACCGGACATGGCACTCCAGTCGCCTTCCCGTTCCGCTATCGG
CTGAATTTGATTGCGAGTGAGATATTTATGCCAGCCAGCCAGACGCAGACGCGC
CGAGACAGAACTTAATGGGCCCGCTAACAGCGCGATTTGCTGGTGACCCAATGC
GACCAGATGCTCCACGCCCAGTCGCGTACCGTCTTCATGGGAGAAAATAATACT
GTTGATGGGTGTCTGGTCAGAGACATCAAGAAATAACGCCGGAACATTAGTGCA
GGCAGCTTCCACAGCAATGGCATCCTGGTCATCCAGCGGATAGTTAATGATCAGC
CCACTGACGCGTTGCGCGAGAAGATTGTGCACCGCCGCTTTACAGGCTTCGACGC
CGCTTCGTTCTACCATCGACACCACCACGCTGGCACCCAGTTGATCGGCGCGAGA
TTTAATCGCCGCGACAATTTGCGACGGCGCGTGCAGGGCCAGACTGGAGGTGGC
AACGCCAATCAGCAACGACTGTTTGCCCGCCAGTTGTTGTGCCACGCGGTTGGGA
ATGTAATTCAGCTCCGCCATCGCCGCTTCCACTTTTTCCCGCGTTTTCGCAGAAAC
GTGGCTGGCCTGGTTCACCACGCGGGAAACGGTCTGATAAGAGACACCGGCATA
CTCTGCGACATCGTATAACGTTACTGGTTTCACATTCACCACCCTGAATTGACTCT

CTTCCGGGCGCTATCATGCCATACCGCGAAAGGTTTTGCGCCATTCGATGGTGTC
CGGGATCTCGACGCTCTCCCTTATGCGACTCCTGCATTAGGAAGCAGCCCAGTAG
TAGGTTGAGGCCGTTGAGCACCGCCGCCGCAAGGAATGGTGCATGCAAGGAGAT
GGCGCCCAACAGTCCCCCGGCCACGGGGCCTGCCACCATACCCACGCCGAAACA
AGCGCTCATGAGCCCGAAGTGGCGAGCCCGATCTTCCCCATCGGTGATGTCGGC
GATATAGGCGCCAGCAACCGCACCTGTGGCGCCGGTGATGCCGGCCACGATGCG
TCCGGCGTAGAGGATCGA
