# Supplementary material for: Unwinding of a eukaryotic origin of replication visualized by cryo-EM
Source: Nat Struct Mol Biol. 2024 May 17;31(8):1265–76. doi: 10.1038/s41594-024-01280-z (PMC11327109; doi:10.1038/s41594-024-01280-z)
Supplement: Supplementary file 2 — Reporting Summary [file 41594_2024_1280_MOESM2_ESM.pdf]

nature portfolio

Corresponding author(s): Alessandro Costa

Last updated by author(s): Mar 5, 2024

Reporting Summary

Nature Portfolio wishes to improve the reproducibility of the work that we publish. This form provides structure for consistency and transparency in reporting. For further information on Nature Portfolio policies, see our Editorial Policies and the Editorial Policy Checklist.

Statistics

For all statistical analyses, confirm that the following items are present in the figure legend, table legend, main text, or Methods section.

n/a	Confirmed	
☐	☒	The exact sample size (*n*) for each experimental group/condition, given as a discrete number and unit of measurement
☐	☒	A statement on whether measurements were taken from distinct samples or whether the same sample was measured repeatedly
☒	☐	The statistical test(s) used AND whether they are one- or two-sided<br>*Only common tests should be described solely by name; describe more complex techniques in the Methods section.*
☒	☐	A description of all covariates tested
☒	☐	A description of any assumptions or corrections, such as tests of normality and adjustment for multiple comparisons
☐	☒	A full description of the statistical parameters including central tendency (e.g. means) or other basic estimates (e.g. regression coefficient) AND variation (e.g. standard deviation) or associated estimates of uncertainty (e.g. confidence intervals)
☐	☒	For null hypothesis testing, the test statistic (e.g. *F*, *t*, *r*) with confidence intervals, effect sizes, degrees of freedom and *P* value noted<br>*Give P values as exact values whenever suitable.*
☒	☐	For Bayesian analysis, information on the choice of priors and Markov chain Monte Carlo settings
☒	☐	For hierarchical and complex designs, identification of the appropriate level for tests and full reporting of outcomes
☒	☐	Estimates of effect sizes (e.g. Cohen's *d*, Pearson's *r*), indicating how they were calculated

Our web collection on statistics for biologists contains articles on many of the points above.

Software and code

Policy information about availability of computer code

Data collection	Gatan DigitalMicrograph, ThermoFisher EPU v2.9 and Monolith NT.115
Data analysis	Topaz v0.2.4, MotionCor2, Ctffind v4.1.13, RELION v3.1, cryoSPARC 4.0, pyem v0.4, bsoft v2.0.4, UCSF Chimera v1.14, ChimeraX-1.3, COOT v0.9-pre, ISOLDE, Phenix v1.20.1, MolProbity web sever, ImageJ2/Fiji v2.3.0, GraphPad Prism v9.4.1

For manuscripts utilizing custom algorithms or software that are central to the research but not yet described in published literature, software must be made available to editors and reviewers. We strongly encourage code deposition in a community repository (e.g. GitHub). See the Nature Portfolio guidelines for submitting code & software for further information.

Data

Policy information about availability of data

All manuscripts must include a data availability statement. This statement should provide the following information, where applicable:

- Accession codes, unique identifiers, or web links for publicly available datasets
- A description of any restrictions on data availability
- For clinical datasets or third party data, please ensure that the statement adheres to our policy

Cryo-EM density consensus maps of the CMGE10 complex has been deposited in the Electron Microscopy Data Bank (EMDB) under the accession number EMD-17459. Cryo-EM density map of ssDNA CMGE10 map has been deposited in the EMDB under the accession number EMD-17458. Cryo-EM density map of nexus-CMGE10 map has been deposited in the EMDB under the accession number EMD-17449. Atomic coordinates have been deposited in the Protein Data Bank (PDB) with the accession numbers 8P63 (consensus CMGE), 8P62 (ssDNA-CMGE) and 8P5E (nexus-CMGE on dsDNA).

Field-specific reporting

Please select the one below that is the best fit for your research. If you are not sure, read the appropriate sections before making your selection.

☒ Life sciences ☐ Behavioural & social sciences ☐ Ecological, evolutionary & environmental sciences

For a reference copy of the document with all sections, see nature.com/documents/nr-reporting-summary-flat.pdf

Life sciences study design

All studies must disclose on these points even when the disclosure is negative.

Sample size	In our negative stain EM experiments, we imaged Mcm10-dependent CMG activation, yielding different reaction intermediates. To isolate CMGs, we usually collected 50-300 micrographs per condition. The sample size was sufficient to either allow 2D classification or comparative analysis between MCM or Mcm10 mutants. All these experiments were performed three times.<br><br>To obtain high-resolution structure of the ssDNA- or dsDNA-bound CMGE in complex with Mcm10, ~71.1 K micrographs were collected from two independent grids made from the same CMG activation reaction. This sample size was appropriate and sufficient to allow model building or comparative analysis.<br><br>No statistical methods were used to predetermine sample size.
Data exclusions	Negative stain and cryo-EM micrographs with poor staining or ice contamination, respectively, were excluded. Picked particles that did not align to a distinct class in 2D and 3D (cryo-EM only) were excluded from further analysis.
Replication	The cryo-EM dataset of Mcm10-dependent CMG activation reaction comprised of a single reaction and two datasets, collected on two independent grids. CMGE10 complex formation in negative stain EM experiments was found to be reproducible across multiple independent sample preparations. Details of the number of experimental repeats have been acknowledged in the relevant figure legends. Details of the number of experimental repeats have been acknowledged in the relevant figure legends. All attempts at data replication were successful.
Randomization	For calculation of the resolution of the cryo-EM reconstructions, Fourier shell correlations were calculated using independent halves of the complete datasets, into which the component particles were segregated randomly.
Blinding	Blinding is not relevant for a single particle electron microscopy study such as this. No risk of bias identifiable.

Reporting for specific materials, systems and methods

We require information from authors about some types of materials, experimental systems and methods used in many studies. Here, indicate whether each material, system or method listed is relevant to your study. If you are not sure if a list item applies to your research, read the appropriate section before selecting a response.

Materials & experimental systems

n/a	Involved in the study	
☒	☐	Antibodies
☐	☒	Eukaryotic cell lines
☒	☐	Palaeontology and archaeology
☒	☐	Animals and other organisms
☒	☐	Human research participants
☒	☐	Clinical data
☒	☐	Dual use research of concern

Methods

n/a	Involved in the study	
☒	☐	ChIP-seq
☒	☐	Flow cytometry
☒	☐	MRI-based neuroimaging

Eukaryotic cell lines

Policy information about cell lines

Cell line source(s)	S. cerevisiae overexpression strains for CMG assembly and DNA replication proteins have previously been described in multiple studies across several publications. For clarity to the potential readers and reviewers we have included extensive details in extended data table X.
Authentication	S. cerevisiae overexpression strains were checked for correct plasmid integration by PCR amplification from extracted genomic DNA.
Mycoplasma contamination	S. cerevisiae overexpression strains were not tested for mycoplasma contamination.

Commonly misidentified lines<br>(See ICLAC register)	No commonly misidentified cell lines were used in this study.
