# Supplementary material for: Unwinding of a eukaryotic origin of replication visualized by cryo-EM
Source: Nat Struct Mol Biol. 2024 May 17;31(8):1265–76. doi: 10.1038/s41594-024-01280-z (PMC11327109; doi:10.1038/s41594-024-01280-z)

Figure 4e

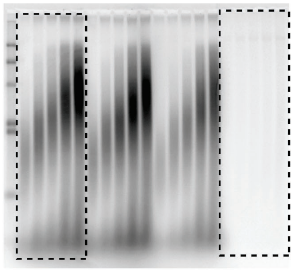

Figure 4f

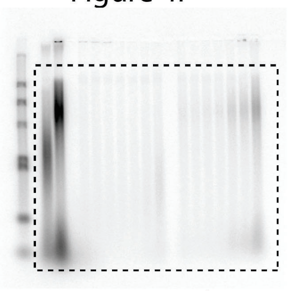

Figure 6c

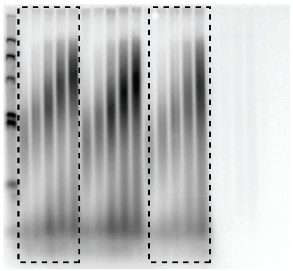

Figure 6d

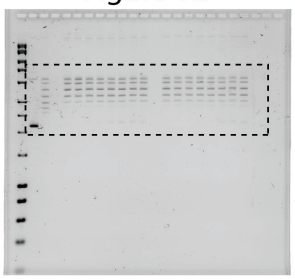

Figure 6e

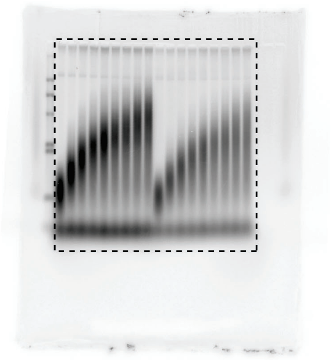

Extended data figure 1a

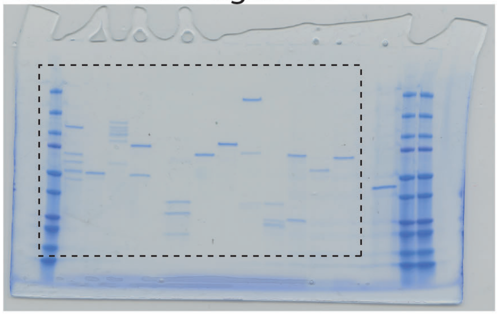

Extended data figure 1b

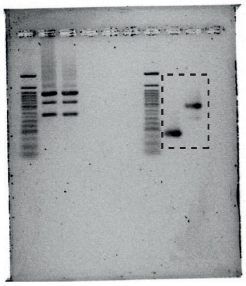

Extended data figure 1a

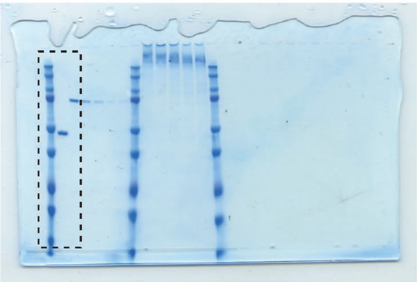

Extended data figure 1a

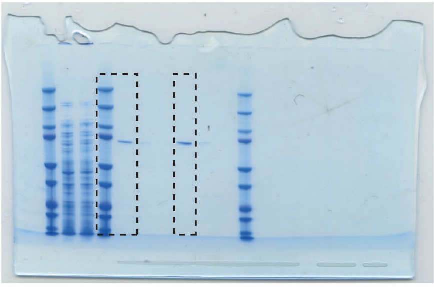

Extended data figure 4d

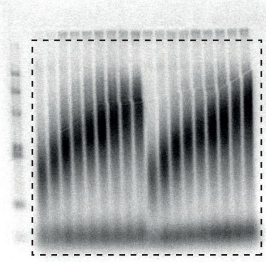

Extended data figure 5b

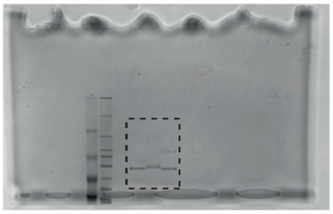

Extended data figure 5d

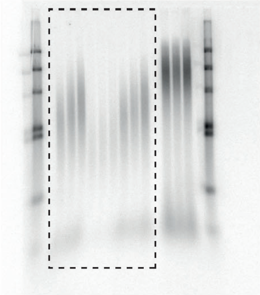

Extended data figure 5e

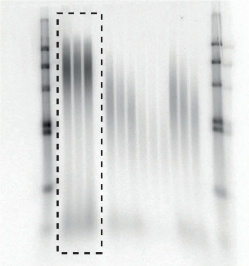

Extended data figure 7a

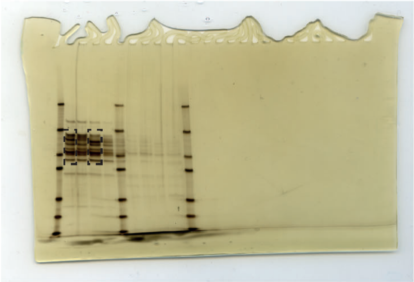

Extended data figure 7d

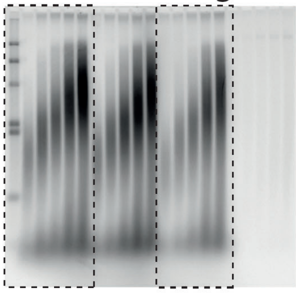

Extended data figure 7e

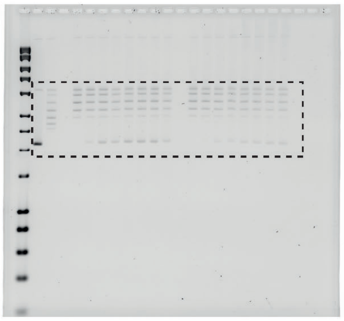

Supplement: Supplementary file 7 — Source images for all data obtained by electrophoretic separation in the figures indicated. [file 41594_2024_1280_MOESM7_ESM.pdf]
